# Gradual and contingent evolutionary emergence of leaf mimicry in butterfly wing patterns

**DOI:** 10.1186/s12862-014-0229-5

**Published:** 2014-11-25

**Authors:** Takao K Suzuki, Shuichiro Tomita, Hideki Sezutsu

**Affiliations:** Transgenic Silkworm Research Unit, Genetically Modified Organism Research Center, National Institute of Agrobiological Sciences, 1-2 Oowashi, 305-8634 Tsukuba, Ibaraki Japan

**Keywords:** Masquerade, Butterfly wing pattern, Phylogenetic comparative methods, Nymphalid ground plan

## Abstract

**Background:**

Special resemblance of animals to natural objects such as leaves provides a representative example of evolutionary adaptation. The existence of such sophisticated features challenges our understanding of how complex adaptive phenotypes evolved. Leaf mimicry typically consists of several pattern elements, the spatial arrangement of which generates the leaf venation-like appearance. However, the process by which leaf patterns evolved remains unclear.

**Results:**

In this study we show the evolutionary origin and process for the leaf pattern in *Kallima* (Nymphalidae) butterflies. Using comparative morphological analyses, we reveal that the wing patterns of *Kallima* and 45 closely related species share the same ground plan, suggesting that the pattern elements of leaf mimicry have been inherited across species with lineage-specific changes of their character states. On the basis of these analyses, phylogenetic comparative methods estimated past states of the pattern elements and enabled reconstruction of the wing patterns of the most recent common ancestor. This analysis shows that the leaf pattern has evolved through several intermediate patterns. Further, we use Bayesian statistical methods to estimate the temporal order of character-state changes in the pattern elements by which leaf mimesis evolved, and show that the pattern elements changed their spatial arrangement (e.g., from a curved line to a straight line) in a stepwise manner and finally establish a close resemblance to a leaf venation-like appearance.

**Conclusions:**

Our study provides the first evidence for stepwise and contingent evolution of leaf mimicry.　 Leaf mimicry patterns evolved in a gradual, rather than a sudden, manner from a non-mimetic ancestor. Through a lineage of *Kallima* butterflies, the leaf patterns evolutionarily originated through temporal accumulation of orchestrated changes in multiple pattern elements.

**Electronic supplementary material:**

The online version of this article (doi:10.1186/s12862-014-0229-5) contains supplementary material, which is available to authorized users.

## Background

Evolution of complex adaptive features is a fundamental subject in evolutionary biology [1-4]. Central questions in relation to this subject include whether the origin of complex features was gradual or sudden, and how the evolutionary changes that generated these features accumulated over long time periods [5-9]. Leaf mimicry in butterfly wings (e.g. genus *Kallima*) provides a striking example of complex adaptive features and has led to speculation about how wing patterns evolve a close resemblance to leaves from an ancestral form that did not resemble leaves [10-13]. Conflicting perspectives on the evolution of leaf mimicry have led to controversial and contrasting hypotheses [14-19]. The origin of leaf mimicry and the process by which it evolved have not been resolved.

**Figure 1:**
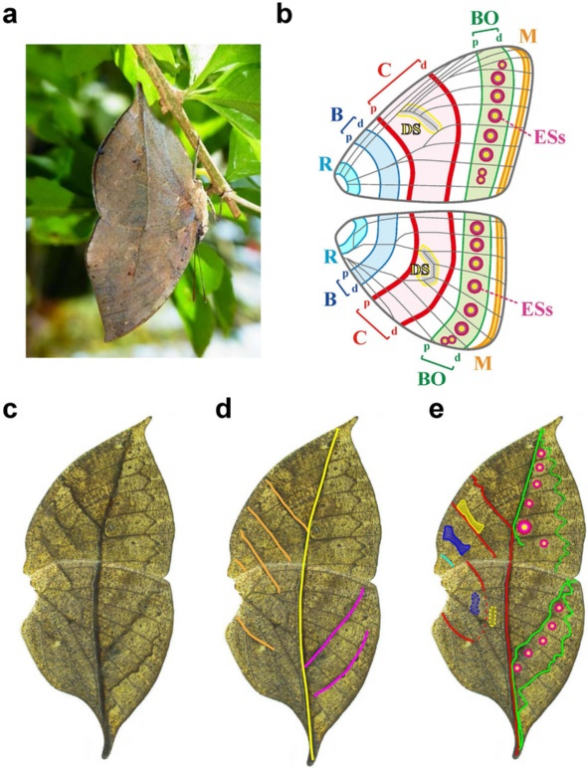
**Nymphalid ground plan and** ***Kallima inachus*** **leaf wing pattern. (a)** When resting, *K. inachus* folds its fore- and hind wings and displays a leaf-like pattern to potential predators. **(b)** Nymphalid ground plan: This scheme consists of 11 elements. The three pairs of symmetry pattern elements include the proximal (p) and distal (d) bands designated as basal (B, blue), central (C, red), and border (BO, green) elements. Four additional elements are designated as root (R, light blue), submarginal and marginal (M, orange), discal spots (DS, yellow) and a serial array of eye spots (ESs, concentric rings). **(c)** Male ventral wings, resembling transverse leaf venation across fore and hind wings. **(d)** The leaf venation pattern is composed of several pattern elements representing a main vein and right and left lateral veins (highlighted with yellow, orange, and pink lines, respectively). **(e)** The Nymphalid ground plan of the *K. inachus* leaf pattern analysed in this study.

**Figure 2:**
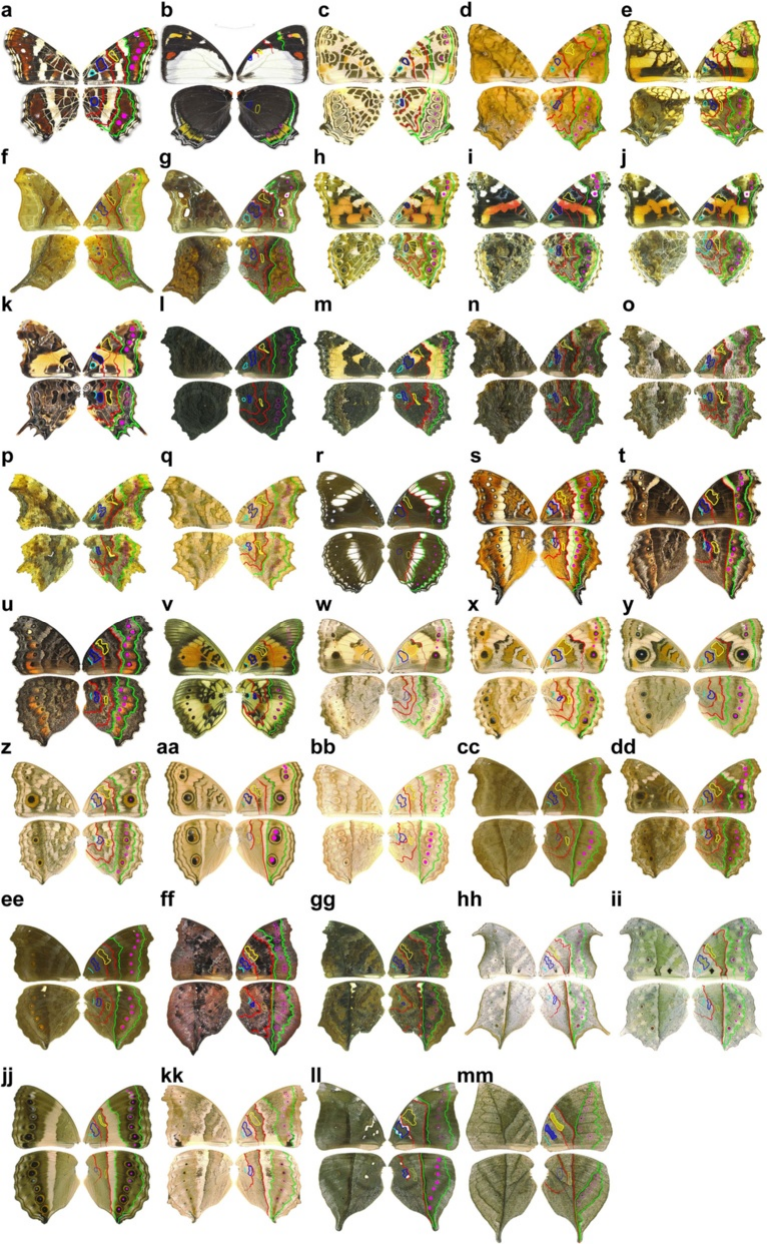
**Nymphalid ground plan of Nymphalinae butterfly wing patterns.** Using a comparative morphological approach, we dissected the extraordinarily diversified wing patterns into an assembly of Nymphalid ground plan (NGP) elements. The right, ventral wings are shown (left, each) with drawings of the NGP (right, each; mirror-opposite images of right wings). **(a)** *Araschnia levana*, **(b)** *Mynes geoffroyi*, **(c)** *Symbrenthia hypselis*, **(d)** *Symbrenthia lilaea*, **(e)** *Hypanartia lethe*, **(f)** *Hypanartia dione*, **(g)** *Hypanartia kefersteini*, **(h)** *Vanessa cardui*, **(i)** *Vanessa atalanta*, **(j)** *Vanessa indica*, **(k)** *Antanartia delius*, **(l)** *Aglais io*, **(m)** *Aglais urticae*, **(n)** *Kaniska canace*, **(o)** *Nymphalis vau-album*, **(p)** *Polygonia c-album*, **(q)** *Polygonia c-aureum*, **(r)** *Hypolimnas bolina*, **(s)** *Precis andremiaja*, **(t)** *Precis archesia*, **(u)** *Precis octavia*, **(v)** *Junonia westermanni*, **(w)** *Junonia hierta*, **(x)** *Junonia orithya*, **(y)** *Junonia coenia*, **(z)** *Junonia lemonias*, **(aa)** *Junonia almana*, **(bb)** *Junonia atlites*, **(cc)** *Junonia iphita*, **(dd)** *Junonia erigone*, **(ee)** *Junonia hedonia*, **(ff)** *Salamis anteva*, **(gg)** *Salamis cacta*, **(hh)** *Protogoniomorpha anacardii*, **(ii)** *Protogoniomorpha parhassus*, **(jj)** *Yoma algina*, **(kk)** *Yoma sabina*, **(ll)** *Doleschallia bisaltide*, **(mm)** *Kallima paralekta*. Colours are the same as those used in Figure 1.

The genus *Kallima* comprises leaf butterflies that display transverse, leaf-like venation across the ventral sides of the fore- and hindwing (Figure 1a, c, d, and Figure 2 mm). The leaf pattern consists of a main vein and right- and left-sided lateral veins, each of which contain pigment elements whose spatial arrangement generates the leaf-like appearance (i.e. pigments, rather than wing veins, form the leaf-like pattern). Leaf mimicry in *Kallima* spp. (*Kallima inachus* and *Kallima paralekta*) was described by Wallace as ‘the most wonderful and undoubted case of protective resemblance in a butterfly’ [14]. Following this description, Darwin, Poulton, and modern evolutionary biologists have argued that the leaf mimicry pattern is a product of gradual evolution by natural selection [10,15-17]. In contrast, Mivart pointed out that although leaf mimicry is assumed to be an evolutionary adaptation, its chance of establishing in a population is predicted to be low because poor mimicry of a target during the incipient stages of evolution would lead to an increased probability of predation [18]. Goldschmidt advocated the sudden emergence of leaf mimicry patterns (i.e. saltation) without intermediate forms [19]. Despite enthusiastic debate, there is as yet no direct experimental evidence for the gradual evolution of the leaf pattern.

We focused on the phylogenetic evolution of leaf mimicry patterns, for which a key principle is the ‘body plan’ or ‘ground plan’, referring to the structural composition of organisms by homologous elements shared across species [20]. Notably, butterfly wing patterns are thought to be based on a highly conserved ground plan (the Nymphalid ground plan, NGP; Figure 1b) [21-23]. The NGP describes the extraordinary diversification of wing patterns as modifications of an assembly of discrete pattern elements shared among species, which are suggested to be homologous and inherited across species. Previous studies have suggested the existence of the NGP in numerous species [23], including the wing patterns of leaf moths [24] and *Kallima inachus* [22]. The NGP has also been validated by experimental molecular data [25]. If the NGP was present in both leaf mimics and non-mimetic butterflies, this would provide an opportunity to examine the evolution of leaf mimicry from non-mimetic patterns by tracing changes in the states of NGP elements through phylogeny.

The identification of homology provides a foundation for statistical testing of the likelihood of trait evolution within a phylogenetic framework. We employed Bayesian phylogenetic inference using BayesTraits [26], which provides a platform for reconstructing ancestral states of traits [27] and for analysing the dependent evolution of state transitions [28]. Furthermore, given the rates of state transitions in traits, it is possible to assess whether changes in one trait are contingent upon the background state of another. In this analysis, contingency was defined as temporal dependency in trait evolution [29-31] and quantified (using the *Z*-score) as the degree of influence of unique, chance historical events on subsequent evolution [*sensu* Pagel [28,32,33]]. Recent studies have documented well-supported molecular phylogeny of *Kallima* and closely related species (tribes Nymphalini, Junoniini, and Kallimini) [34-36], which facilitates Bayesian phylogenetic inference.

Our objectives were to generate statistical estimation of (1) ancestral wing patterns given a lineage of leaf mimicry evolution, and (2) evolutionary process of accumulation in state changes of NGP elements. Through these analyses, we examined whether leaf mimicry evolved through gradual or sudden changes and whether these changes accumulated independently or contingently. Here, we show the evolutionary origin and process of the *Kallima* leaf pattern. We demonstrate that the leaf pattern is composed of an array of discrete elements described by the NGP that are also present in the wing patterns of closely related species. These results strongly suggest that evolution of the *Kallima* leaf pattern can be traced by changes in the states of NGP elements. We then use Bayesian phylogenetic methods to reconstruct ancestral wing patterns, and describe the evolution of leaf patterns through stepwise changes in intermediate states from the non-mimetic ancestral pattern.

## Methods

### Sampling strategy

**Figure 3:**
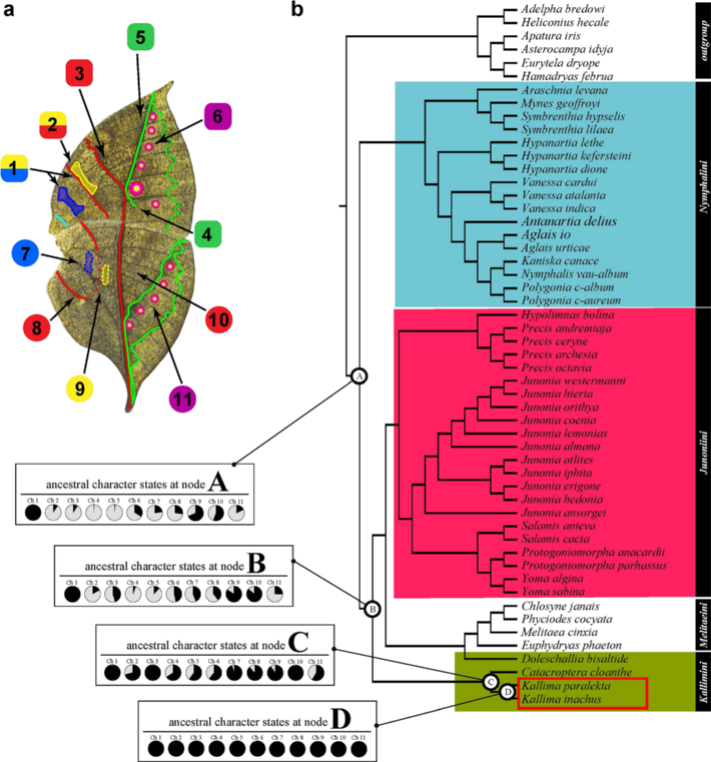
**Bayesian inference of ancestral character** *-* **state reconstruction of wing pattern evolution. (a)** Leaf vein features of the *Kallima* wing pattern were coded as 11 characters (Ch) as follows: Ch 1: parallelism of DS and B; Ch 2: attachment of DS and Cp; Ch 3: Cd a single broken straight line; Ch 4: bending of BOp to distal side; Ch 5: straightness of upper side of BOp; Ch 6: vestigiality of ESs; Ch 7: vestigiality of B; Ch 8: fragmentation of Cp; Ch 9: vestigiality of DS; Ch 10: straightness of Cd; Ch 11: vestigiality of ESs. These characters were also surveyed in the closely related species and coded as one of two binary states (‘state 1’ = *Kallima*-like state; ‘state 0’ = non-*Kallima*-like state). Characters in forewings (squares) and hindwings (circles) are coloured as in Figure 1. **(b)** Reconstructed ancestral character states are represented as pie charts indicating Bayesian posterior probability at four selected nodes (A, B, C, and D) by shaded circles (black = state 1; grey = state 0). In the molecular phylogeny, genus *Kallima* is highlighted in the red box. B, Cp, Cd, BOp, DS, and ESs are defined and presented in Figure 1.

The species used in this study were selected to represent major groups of Nymphalinae, which includes three higher taxa (Kallimini, Junoniini, Nymphalini). Among all genera (22 genera) comprising these three higher taxa, we selected 18 genera (Additional file 1: Figure S1). Among all species (196 species) comprising these 18 genera, we sampled 47 species (24%) (Additional file 1: Table S1). In the analyses, one major group of Nymphalinae, Melitaeini, was excluded because of very autapomorphic wing patterns [36-38], except for the following 4 species from 4 genera: *Euphydryas phaeton, Melitaea cinxia*, *Phyciodes cocyta*, and *Chlosyne janais*. Phylogenetic comparative methods assume that extant species are either completely or proportionally sampled from the taxon of interest. We thus intended to minimize the effects of biased sampling on our statistical inferences by selecting representative species sampled from almost all genera. To evaluate whether the species we selected are representative of their genus with regard to wing patterns, we checked photos of butterfly wing patterns from validated and private web sites (Additional file 2: Table S2). Because our analyses focus on geometrical characteristics (e.g., a straight line and parallel arrangement between lines) of pigmental elements forming wing patterns (Figure 3a), it is necessary to select species displaying representative wing patterns in the genus that the species belong to. Therefore, we observed the specimens and photos to determine whether the 11 characteristic states of the NGP used for phylogenetic comparative analyses are typical of the genera. We checked 116 species (89% of all 131 species) and confirmed the unbiased selection of the species used in this study. For example, in the genus *Kallima*, the two species we selected (*Kallima inachus* and *paralekta*) appeared to be representative to this genus because they exhibited wing patterns similar with to those of another species (*Kallima alompra*) with regard to the Nymphalid ground plan (NGP; see Figures 1b, 3a) (Additional file 1: Figure S2). Thus, although the results should be interpreted cautiously, we are confident that by applying unbiased sampling of species from most genera, we conducted a practical estimation of the evolution of wing patterns.

### Molecular phylogenetic analysis

To take phylogenetic and branch-length uncertainty into account in our analyses, we generated Bayesian trees by combining three recently published datasets [34,36,39] and confirmed that our phylogeny was consistent with that proposed previously (Additional file 1: Figure S3). We used eight nuclear (*wingless*, *ef-1α*, *RpS5*, *GADPH*, *ArgKin*, *CAD*, *IDH* and *MDH*) and one mitochondrial (*cox1*) gene sequences to reconstitute the phylogenetic tree of the species included in the analysis. Multiple alignment was performed using ClustalW [40] in MEGA5 [41] as previously described [42]. In brief, we aligned the nucleotide sequences based on their translated amino acid sequences, and the aligned sets of genes were concatenated for use in subsequent analyses. Species names and GenBank accession numbers of sequences used in this study are provided in Additional file 1: Table S3. The original images of voucher specimens are cited in the NSG’s DNA sequences database (http://nymphalidae.utu.fi/db.php). Six species (*Adelpha bredowii*, *Apatura iris*, *Asterocampa idyja*, *Eurytela dryope*, *Hamadryas februa*, and *Heliconius hecale*) were used as the outgroup taxa. We constructed datasets composed of 7,342 nucleotide sites from nine concatenated genes.

We used PartitionFinder [43] to identify nucleotide substitution models and partitioning strategies for the dataset. Breaking down the nucleotide data by codon position resulted in 27 partitions (the first, second, and third codon positions for each gene), which were combined to result in nine partitions. A nucleotide substitution model was selected for each partition using the number of sites as the sample size based on the Bayesian information criterion (BIC) (see Additional file 3: Data S1: the attached nexus file for the alignment, partitioning and substitution models). The sequence data as well as phylogenetic analysis are also available at TreeBASE (Submission ID: 16541). We used MrBayes 3.1.2 for the Bayesian inference of phylogenetic trees, which includes the assumption of proportional branch length among the partitions. We ran four concurrent analyses of 2 × 10^7^ generations with eight chains each (seven heated and one cold) using different random starting trees, and sampled every 100 generations. Runs of all procedures were checked for stationarity, convergence, and adequate mixing of the Markov chains using Tracer version 1.5 [44]. From each data set, we discarded the first 60,000 samplings as burn-in and combined the resulting MCMC tree samples for subsequent estimation of posteriors.

### Comparative morphological analysis for character assignment

Identification of the NGP elements was conducted based on Remane’s criteria, a validated comparative morphological procedure used in systematic studies [45]. This criterion consists of three principal rules: 1) similarity of topographical relationships, 2) similarity of special features, and 3) transformational continuity through intermediate ontogeny or phylogeny. We used the first and second criteria in this study. The first criterion is logically consistent with Geoffroy St. Hilaire’s ‘*principe des connexions*’ [46]. To use this criterion, it is necessary to set the fixed point of the topographical frame, which is explicitly referenced by Rieppel [47]. The position of the discal cell (a unique feature of wing veins) is suggested for use as the fixed point for investigations of butterfly wing patterns [23,24]. The identification procedure was performed as follows: (i) using the discal cell as a fixed point, the DS and B (located at distal and middle sites in the discal cell, respectively) are easily identified; (ii) according to the topographical relationships among elements, the Cp was identified between the DS and B; (iii) unique concentric shapes (Remane’s second criterion) formed a series of ESs; (iv) the Cd and BOp were arrayed in an orderly fashion from the DS to ESs; and (v) BOd was identified at the outer location of the ESs. To confirm the identification of the NGP elements, we observed several specimens and used photographs from the NSG’s DNA sequence database (http://nymphalidae.utu.fi/db.php). Although some species were referenced in previous studies [21-23], there was no previous foundation for their validation. We used this rigorous method to identify the NGP; to clearly relate the positions of morphological elements to features of the wing veins, pigment cells surrounding the wing surface were carefully removed (except for *Precis ceryne, Junonia ansorgei, Catacroptera cloanthe*). Although the NGP of the wing patterns was rigorously identified by a comparative morphological procedure, such determinations of homology remain hypothetical because of the lack of developmental genetic data.

### Character coding

Morphological characteristics of butterfly wing patterns have been coded based on the NGP and systematic observations of individual specimens [48,49]. Because the present study specifically focused on the evolution of leaf mimicry patterns in *Kallima* spp., character coding should be performed relative to the morphological characteristics of this species wing pattern. Therefore, we coded the morphology of wing-pattern elements as one of two states: 0 (not *Kallima inachus*-like) or 1 (*Kallima inachus*-like). This coding strategy, where all states other than the one of interest are collapsed into a single state (e.g., 0), was previously studied [50]. Eleven morphological characters were coded, of which characters 1–6 were derived from the forewing and characters 7–11 were derived from the hindwing as follows. Character 1: parallelism of DS and B (0 = not parallel, 1 = parallel); character 2: attachment of DS and Cp (0 = not attached, 1 = attached); character 3: Cd consists of a single broken line (0 = not a single, 1 = a single); character 4: BOp bends to the distal side (0 = BOp bends proximally, 1 = BOp bends distally); character 5: straightness of upper side of BOp (0 = upper side of BOp not straight, 1 = upper side of BOp straight); character 6: vestigiality of ESs (0 = ESs not vestigial, 1 = ESs vestigial); character 7: vestigiality of B (0 = B not vestigial, 1 = B vestigial); character 8: fragmentation of Cp (0 = Cp not fragmented, 1 = Cp fragmented); character 9: vestigiality of DS (0 = DS not vestigial, 1 = DS vestigial); character 10: straightness of Cd (0 = Cd not straight, 1 = Cd straight); character 11: vestigiality of ESs (0 = ESs not vestigial, 1 = ESs vestigial). The character codes for the butterfly wing patterns are summarized in Additional file 1: Tables S4 and S5.

### Estimation of common ancestral states at phylogenetic nodes

Reconstruction of ancestral character states was performed in a Bayesian framework using BayesTraits ver. 2.0 (www.evolution.rdg.ac.uk/BayesTraits.html) [26]. In contrast to the optimality criterion (parsimony and likelihood), the Bayesian Markov chain Monte Carlo (MCMC) method has the advantage of investigating the uncertainty of the phylogeny and the parameters of the model for trait evolution [27]. BayesTraits implements the program MULTISTATE, which calculates the posterior probability of states in all nodes across the posterior distribution of trees that are hypothetical ancestors of the taxa of interest. This calculation uses reversible-jump (rj)-MCMC simulations to combine uncertainty about the existence of a node and its character state, which enables sampling of all possible models of evolution (rather than just the rate parameters as in conventional MCMC) in proportion to their posterior probabilities [28,51]. Reconstructions were performed using the most recent common ancestor (MRCA) approach; when the node of interest did not exist, the minimal node that contained all terminal taxa of the clade defined by our node of interest (plus one or more extra taxa) was reconstructed instead. In these analyses, polymorphic character states were accounted for, as they were considered as occurrences with an equivalent probability for calculation [26].

To run the rj-MCMC chain, 4,000 trees were subsampled from each of the four codon-partitioned MrBayes runs (a total of 2 × 10^5^ trees). To allow adequate mixing and achievement of stationary, the rj-MCMC chain was run for 5.005 × 10^7^ iterations with the first 5 × 10^4^ iterations discarded as burn-in and a sampling interval of 1000 iterations, for a final sample of 5 × 10^4^ iterations. We used a uniform prior for the analyses. To avoid autocorrelation and to allow exploration of ample parameter space, the ratedev parameter was automatically adjusted for each analysis to maintain an acceptance rate of 30%, to vary the amount by which the rate parameters were allowed to change between iterations of the Markov chain (ratedev), as recommended in the BayesTraits manual [26]. We examined the output in Tracer version 1.5 [44] to confirm the stationarity of the log-likelihood. Manipulation of trees was conducted using the ‘ape’ package [52] in R.

### Estimation of dependent evolution

We used the DISCRETE program implemented in BayesTraits [26,28] to test for (in)dependence of pairs of character state changes. As described above, this method also controls for uncertainty of phylogeny and model parameters for trait evolution [28]. The BayesDiscrete model describes changes in two dichotomous traits over branches of a phylogenetic tree via a continuous-time Markov process. The parameters of the trait evolution model represent the values of the transition rates between the eight possible character states in a model of dependent evolution. Eight rate parameters constitute the dependent model, which assumes that each character evolves (shifts forwards and backwards) at different rates depending on the state of the second character. In the independent model, forward and backward shifts in one character occur at the same rate regardless of the state of the second parameter (i.e. q_12_ = q_34_, q_13_ = q_24_, q_21_ = q_43_, and q_31_ = q_42_). Hence, a model of independent evolution has four parameters. The dependence of two traits can be investigated by comparing an independent model (in which traits evolve independently) with a dependent model where traits evolve in a correlated fashion. These models can be compared from the logarithmic Bayes Factor (log-BF), calculated as [2(log[harmonic mean (dependent model)] – log[harmonic mean (independent model)]) [26]. The harmonic means of the log-likelihoods converge on the marginal likelihoods after an adequately long run of the Markov chain [53], and therefore can be used in calculating the Bayes factors. Following Pagel and Meade [26], a result of greater than 2 approximated by the harmonic means from the final iteration of the MCMC runs was considered to represent evidence favouring the dependent model (Additional file 1: Table S6).

We used the same trees and settings (rj-MCMC and ratedev) for the Bayesian analysis of dependent evolution as were used for estimation of common ancestral states at phylogenetic nodes. We ran 5.005 × 10^8^ generations, sampling every 1000 generations to yield a sample of 5 × 10^5^ iterations after 5 × 10^5^ iterations were removed as burn-in. We used the same prior (a uniform prior or a gamma hyperprior) for comparison of the harmonic means of the independent and dependent models for each pair of character state changes. Because of potential instability of the harmonic mean likelihood [28,53], we performed at least three separate MCMC runs for each analysis to ensure stability within and among runs.

### Estimation of order of accumulation

The posterior probability distributions of the eight transition parameters in the dependent evolutionary model were used to estimate whether the change in one state was contingent upon the background state of the other state [26,28]. This calculation was made from the proportion of evolutionary models analysed by rj-MCMC for which a value of zero was assigned to the transition parameter (*Z*-score) [28,32,33]. If the value of one transition rate parameter in the dependent model shows a higher *Z*-score, that transition is less likely to occur. Thus, the *Z*-score represents a degree of restriction, where evolutionary trajectories or pathways are constrained. Contingency of evolutionary changes was evaluated by comparing critical pairs of parameters (i.e. q_12_ vs. q_34_; q_13_ vs. q_24_). For example, if q_12_ (the rate parameter for the [0, 0] → [0, 1] transition) shows a higher *Z*-score, but q_34_ (the rate parameter for the [1, 0] → [1, 1] transition) shows a lower *Z*-score, then evolutionary change of the second character from 0 to 1 is more likely to occur when the background state of the first character =1. This evolutionary case can be interpreted to indicate that change in the second character is contingent upon change in the first character. In the Bayesian phylogenetic method, contingency in changes in character states was detected by a bias between sets of two transition rates (Additional file 1: Table S7 and Figure S4).

**Figure 4:**
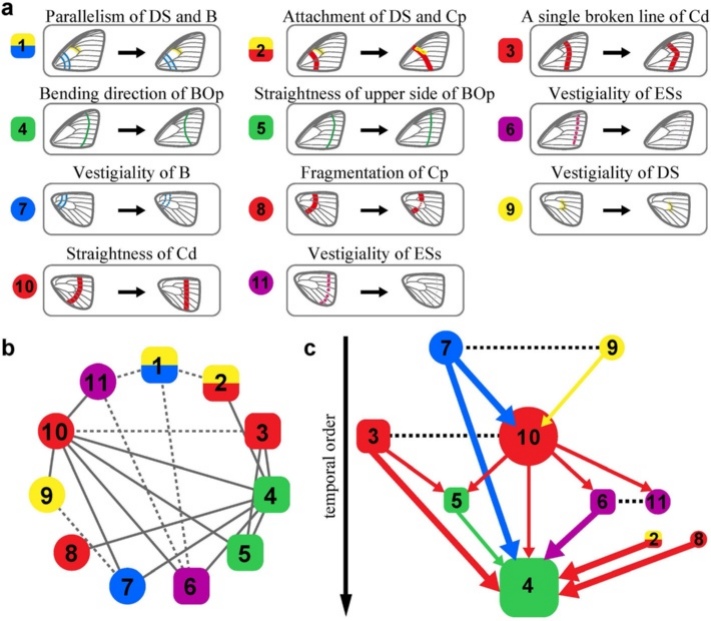
**Bayesian inference of temporal order in character state changes. (a)** Evolutionary changes in 11 characters from state ‘0’ to state ‘1’. **(b)** Evolutionary dependency map: Summary of dependent evolution between character state changes. Mutual dependency is shown by dotted lines, whereas contingent dependency by solid. **(c)** Evolutionary contingency network: Temporal order of accumulation of character state changes. Line thickness reflects Z-score (thin lines for lower Z-scores (>70%) and thick for higher Z-scores (>90%)). Earlier changes are presented at the top of the network. Mutually dependent evolution is shown by dotted lines (no arrow). Characters in forewings (squares) and hindwings (circles) are coloured as in Figure 1.

Estimation of contingency was conducted within pairs of character states that showed dependent evolution (Additional file 1: Table S8). Contingency between a pair of character state changes was determined from the results of the model with a gamma hyperprior and evaluated according to relatively low transition rates (*Z*-score >70%) [32,54,55]. The contingency between all pairs of character state changes was summarized in the form of a network (Figure 4c), in which nodes represent events in the changes of character states and arrowed links represent the order of accumulation of character state changes.

## Results

### Identification of the NGP in *Kallima* and closely related butterflies

Using Remane’s criteria [45], a rigorous comparative morphological method, we identified that the *K. inachus* leaf pattern was composed of the elements described by the NGP (Figure 1e). Our analysis decomposed the main vein of the leaf pattern into three NGP elements: the upper side of the border proximal (BOp) element, the lower side of the central distal (Cd) element in the forewing, and the Cd element in the hindwing. The analysis then decomposed the left-sided lateral vein pattern into six NGP elements: upper side of the Cd element, discal spots (DS) and central proximal (Cp) element (closely attached to form a single straight line), basal (B) and root (R) elements in the forewing, and the Cp element (fragmented into two elements) in the hindwing. The right-sided lateral vein pattern was composed of two NGP elements: the BOp and border distal (BOd) element in the hind wing. The eyespots (ESs) in both the fore and hind wings became vestigial. In this analysis, our rigorous method validated the inference proposed by Süffert [22]. We also examined *Kallima paralekta* and revealed that the leaf pattern in this species showed almost identical characteristics to those of *K. inachus* (Figure 2mm). The *Kallima* leaf patterns were thus depicted by an assembly of the NGP elements.

Subsequently, we found that the wing patterns of 45 butterfly species closely related to genus *Kallima* were also composed of the NGP elements, although their appearances differed from the leaf-like patterns found in *Kallima* (Figure 2). Notably, differences among the species could be attributed to differences in geometric shape of the NGP elements. For example, in *Kallima*, the Cd element in the hindwing formed a straight line (Figure 1e and Figure 2 mm), whereas in *Polygonia c-album* it did not (Figure 2p). Collectively, the Cd elements in hindwings formed straight lines in twelve species (summarized in Additional file 1: Table S5, character (Ch) 10; Figure 2). These results strongly suggest that the *Kallima* leaf patterns originated through evolutionary changes in character states of NGP elements from the ancestral species.

### Bayesian phylogenetic inference of ancestral wing patterns

The results of our comparative morphological analyses explain the evolution of the *Kallima* leaf patterns, which are formed of NGP elements with specific modifications that confer a leaf-like appearance. A Bayesian phylogenetic method was used to reconstruct the ancestral states of the butterfly wing patterns at phylogenetic nodes (A–D in Figure 3b). We coded the *K. inachus* wing pattern using 11 prominent characters from the suite of characteristics that formed the leaf-like appearance (Figure 3a). This coding was also performed on the closely related species and their wing patterns were characterized as one of two binary states (Additional file 1: Tables S4 and S5). Analyses implemented in BayesTraits account for uncertainty in phylogeny and branch length; we reconstructed phylogenetic trees by combining three previously published datasets [34,36,39] (Figure 3b; Additional file 1: Figure S3) and obtained results that were consistent with previous reports [34,36,39]. The 11 character states at node A were estimated as follows in the forewing: the Cp and DS elements were not attached (Ch 2), the Cd element did not form a single, broken straight line (Ch 3), the BOp was ordinarily curved (Ch 4 and 5), and the ESs were not vestigial (Ch 6). In the hind wing, the B, DS, and ESs were not vestigial (Ch 7, 9, and 11), the Cp element was not fragmented (Ch 8), and the Cd element did not form a straight line (Ch 10) (Figure 3b). Taken together, these results strongly suggested that the most ancestral pattern was a non *Kallima*-like pattern.

Our analyses revealed further evolution of wing patterns (Figure 3b). At node B of the phylogeny, the character states were reconstructed such that DS in the hindwing became vestigial (Ch 9) and the Cd in the hindwing became straightened (Ch 10). The overall wing pattern evolved through the accumulation of changes from the most ancestral wing pattern at node A. Then, at node C, the Cd in the forewing changed to a single broken line (Ch 3), the B in the hindwing was vestigial (Ch 7), and the Cp element in the hindwing became fragmented (Ch 8). The wing pattern evolved through additional changes that caused some characteristics to transition to a *Kallima*-like state. Finally, at node D, all character states had transitioned to the *Kallima*-like state (state ‘1’). These analyses demonstrate that the overall leaf pattern originated via stepwise transitions through intermediate forms. These results clearly showed that, at the very least, this evolutionary transition did not occur suddenly.

### Evolutionary accumulation of character state changes in evolution of wing patterns

The above analyses revealed that leaf mimicry evolved via stepwise transitions from one intermediate state to another. Evolution by natural selection is expected to progress through the stepwise accumulation of changes. To gain an improved understanding of the evolution of leaf patterns, we further examined the process of accumulation of character state changes of NGP elements, focusing on mutual and temporal dependency (i.e. contingency) of the changes.

Our Bayesian phylogenetic analyses inferred that evolutionary changes in some pairs of character states were interdependent (Figure 4). Among all possible combinations of pairs of characters (*n* =55 combination), 19 pairs of character state changes were significantly supported as dependent (logarithmic Bayes Factor (log-BF) >2; Additional file 1: Table S6); these dependencies were summarized as a linkage map (Figure 4b). Subsequent analyses examined whether cases of dependent evolution consisted of mutual or temporal dependence (Additional file 1: Table S8). Among the 19 pairs of character states showing dependent evolution, six demonstrated mutual dependence and 13 evidenced temporal dependence (*Z*-score >70%; Additional file 1: Table S7 and Figure S4). The temporal dependence indicated that some state changes in NGP elements were contingent upon the background states of other elements, suggesting a temporal order of accumulation of the changes, summarized as a form of network (Figure 4c). The accumulation of character state changes occurred in the following order: (1) independent loss of the hindwing DS and B (Ch 7 and 9); (2) evolution of the hindwing Bd as a straight line (Ch 10) and of the forewing Cd into a single broken straight line (Ch 3); (3) evolution of the upper BOp into a straight pattern (Ch 5); (4) transition of the bend in the hindwing BOp from proximal to distal (Ch 4). On the other hand, after the Cd in the hindwing straightened, the ESs in both fore- and hindwings became vestigial (Ch 6 and 11). Taken together, evolution of the *Kallima* leaf pattern progressed in concert with the sequential accumulation of state changes in NGP elements.

## Discussion

**Figure 5:**
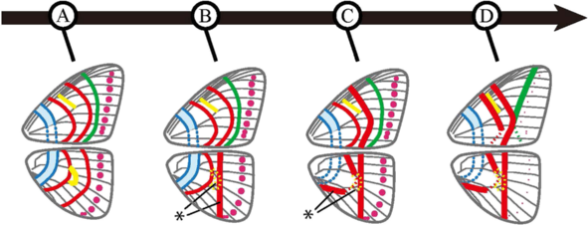
**Schematic illustration of gradual evolution of** ***Kallima*** **leaf mimicry patterns.** The leaf pattern emerged through several intermediate patterns from the non-mimetic ancestor. The historical time course is shown using four time points **(A–D)** that correspond to the phylogenetic nodes in Figure 3. At the most ancestral point, the wing pattern may reflect the original developmental basis of the NGP **(A)**. Finally, the wing pattern reached the *Kallima*-like state **(D)**. Evolutionary changes to the character state ‘1’ are shown at the nodes where a posterior probability of 0.95 or greater occurs (some changes (marked by asterisks) are based on a posterior probability of 0.85 or greater, which represents relatively lower confidence). Characters and NGP elements are coloured as in Figure 1.

This study delivers the first clear picture of the evolution of leaf mimicry in *Kallima* butterflies. Our analyses clarified the ways in which butterfly wing patterns evolve to resemble leaves. From the perspective of comparative morphology, the *Kallima* leaf patterns are decomposed into homologous pattern elements shared across closely related species. A lineage of leaf mimicry patterns evolved through stepwise changes and intermediate wing patterns. These results were further corroborated by in-depth analyses of trait evolution that revealed contingency in the sequential accumulation of traits; changes in states of elements influenced the subsequent evolution of other state changes. Although some have argued that the gradual evolution of leaf mimicry is improbable [18,19], our analyses reveal that leaf mimicry evolved gradually from the non-mimetic pattern without a sudden transition. We conclude that *Kallima* leaf patterns evolved in a stepwise manner through intermediate forms with sequential accumulation of state changes (Figure 5).

When performing phylogenetic comparative methods, it is important to perform an unbiased sampling of species to ensure accurate estimation of ancestral states or contingent evolution. We are confident that our analyses were conducted under satisfactory conditions for several reasons: (1) the species used in this study were selected to represent major groups of Nymphalinae, which was carefully checked by observing photos of species from validated and private web sites (Additional file 2: Table S2); (2) our reconstruction of species trees was consistent with previously reported trees; (3) for coding wing-pattern characters, specimens were carefully collected to identify the position of morphological elements relative to that of the wing veins for Remane’s framework. Given these points, it seems reasonable to consider that our analyses provide a practical estimation of phylogenetic statistical analyses, using a sufficient number and appropriate selection of species.

The temporal order of the accumulation of character state changes was assessed using *Z*-scores. Thus, contingency was a quantitative value and subsequent evolution was influenced to some degree by unique, chance historical events [28,32,33]. In the network of contingent evolution, only four character pairs showed strong evidence of contingent evolution, where the occurrence of one state change was highly contingent upon the background state of the other (*Z*-score >95%; Figure 4c, Additional file 1: Figure S4). The other three pairs of character state changes showed moderate contingency (*Z*-score >90%). Under the loosest criteria (*Z*-score >70%), six pairs showed weak contingency, where one state change loosely restricted subsequent state changes. These results strongly suggested that the evolutionary trajectories in this lineage were not completely restricted. In fact, an alternative temporal order in state changes was likely to have occurred. For example, before the loss of the hindwing B (Ch 7), a straight hindwing Bd (Ch 10) occurred in five species (*Doleschallia bisaltide, Protogoniomorpha anacardii, Protogoniomorpha parhassus*, *Yoma Sabina*, and *Precis archesia*). Although the evolutionary restriction varied from weak to strong, our analyses provided evidence that evolution of the leaf pattern resulted in sequential accumulation of character state changes.

We provide the analytical framework for evolution of complex adaptive phenotypes within a phylogenetic context. Comparative morphological analysis revealed that *Kallima* spp. and closely related species were explained by the same NGP scheme. Thus, the morphological differences between species were attributable to differences in the states of subordinate elements. We suggest that this analytical approach is applicable to the evolution of other types of camouflage (e.g. lichen and tree-bark mimetic patterns). Previous studies demonstrated the utility of phylogenetic approaches to studying the evolution of camouflage and mimicry [56-58], but the advantage of the ground plan scheme was unexplored. The phylogenetic homology-based comparative method used here provides a powerful way to explore evolutionary origins and processes of camouflage and mimicry as well as other morphological evolution, although the extent to which this framework can be applied to other cases remains to be determined.

Although our data strongly suggest that leaf mimicry emerged gradually through intermediate states of wing patterns, the survival mechanisms of butterflies with intermediate patterns remain unclear. To date, many believe that intermediate wing patterns represent poor forms of mimicry, which generates criticism of the possibility of gradual evolution of leaf mimicry [18,19,59,60]. One plausible explanation for survival of poor mimics is found in the concept of imperfect mimicry [61], which maintains that the survival of poor mimics (e.g. hoverflies that are poor wasp mimics [62,63]) is often explained by a trade-off in predator foraging behaviours (e.g. a trade-off between the speed and accuracy of decision-making [64]). This concept suggests the following evolutionary scenario: the larger the area in which a predator seeks prey, the less time the predator has to discriminate whether an object it encounters is edible, the lower the accuracy of discrimination, and the higher the probability that a poor mimic escapes predation [65]. Applying this scenario to poor leaf mimics, predators may misidentify prey as leaves. Because special resemblance of animals to natural objects is termed masquerade, we propose a hypothesis of “imperfect masquerade” for the above phenomena.

According to Darwin’s theory, the sequential accumulation of individual changes is essential for natural selection of fortuitous variation. The present study demonstrated that evolution of leaf mimicry progressed in association with the sequential accumulation of character state changes. Although our analyses were based on phenotypic evolution, these phenotypic changes are likely to be a result of a series of multiple genetic changes fixed by natural selection and neutral drift accumulated over long time periods. Because our comparative morphological analyses demonstrated that the wing patterns of species closely related to Kallima leaf butterflies are explained by the NGP, it is hypothesized that the developmental processes forming the wing patterns of these species include common molecular mechanisms, probably core developmental programs for wing pattern formation to satisfy the NGP. Moreover, our analyses demonstrated that evolutionary divergence of these wing patterns occurs through alteration of the characteristic states of the NGP elements (e.g., the Cd of the hind wing from a non-straight line to a straight line), strongly suggesting that secondary modifications have accumulated in the regulatory process to affect the behavior of such core molecular programs through long-term evolution. These investigations reveal genetic and developmental mechanisms underlying the evolution of leaf mimicry, as previously demonstrated for the molecular mechanisms and evolution of other types of butterfly wing patterns [66-69]. Recent progress in omics approaches enables de novo genome assembly [70] and transcriptome profiling of wildlife [71]. Such analyses will provide great insight into genetic alterations and the relevant developmental molecular mechanisms that cause changes in wing patterns, and will provide further evidence for the stepwise evolution of leaf mimicry patterns.

Leaf mimicry provides a textbook example of adaptation and is also a historically contentious subject that has spurred criticism of modern evolutionary synthesis. Although Darwin and Wallace (and subsequent evolutionary biologists) argued for the gradual origin of leaf mimesis, the lack of direct experimental evidence has allowed antagonists to produce alternative evolutionary scenarios (e.g. saltation) for leaf mimesis [19]. In the past, leaf mimicry in *Kallima* butterfly species was a cornerstone in arguments for saltatory evolution, in which context Goldschmidt argued that this mimicry must have originated suddenly as a ‘hopeful monster’, without any intermediate forms [19]. The discovery revealed in this study refutes such claims and demonstrates that the appearance of leaf mimicry in *Kallima* spp. has arisen in a stepwise and contingent fashion.

## Conclusion

This study delivers the first clear picture of the evolutionary emergence of leaf mimicry in *Kallima* butterflies. The evolutionary emergence of leaf mimicry has been a historically contentious issue and remains an unresolved conundrum. Our analyses resolved this conundrum by demonstrating that the leaf pattern evolved gradually from a non-mimetic pattern. Although we could not show the survival mechanisms of butterflies with intermediate patterns, the results of this study strongly suggest evolutionary trajectories toward leaf mimicry via intermediate states of wing patterns, and we therefore proposed an ‘imperfect masquerade’ to explain the presence of wing patterns with intermediate states. In the future, it will be necessary to investigate how butterflies with such intermediate patterns can survive by investigating the foraging behaviour of predators and escape strategy of butterflies.

In addition, we elaborated a powerful method to explore the evolutionary process of complex adaptive phenotypes. To date, comparative morphological approaches were used to investigate macro-level evolution; however, these approaches could be hardly applied to micro-level evolution, partly because of the lack of appropriate statistical methods to detect subtle phenotypic changes. The method that we developed is based on a comparative morphological approach in combination with phylogenetic Bayesian statistics, which can be applied to a various examples of phenotypic evolution such as the evolution of vertebrate neuro-musculo-skeletal systems or that of insect camouflage and mimicry.

### Availability of supporting data

The data set supporting the results of this article is available in the LabArchives repository at doi:10.6070/H4S180G7.

## Additional files

Additional file 1: Figure S1. Phylogenetic relationship among genera of Kallima, Junoniini, and Nymphalini. Phylogeny of 22 genera of Nymphalinae is shown. **Figure S2**. Comparison of leaf wing patterns among three species of the genus Kallima. The ventral fore and hind wings of three species of Kallima are shown. **Figure S3**. Phylogenetic tree for Nymphalinae based on nine protein-coding gene sequences. The phylogenetic tree inferred by Bayesian analysis is depicted with branch lengths proportional to time. **Figure S4**. Contingent order detected as a bias of the transition rate using the Z-score. Contingency was analysed by comparing specific sets of transition parameters and the corresponding Z-scores. **Table S1**. Sampling density of species in this study. The genera of Nymphalinae and the number of species within the genera are described. **Table S3**. Species and Genbank accession numbers of the nine genes used. Eight nuclear (wingless, ef-1α, RpS5, GADPH, ArgKin, CAD, IDH and MDH) and one mitochondrial (cox1) gene accession IDs were listed. **Table S4**. The character states of Nymphalinae ventral-sided forewing patterns based on the Nymphalid ground plan used in this study. Character coding of forewing of species analysed. **Table S5**. The character states of Nymphalinae ventral-sided hindwing patterns based on the Nymphalid ground plan used in this study. Character coding of hindwing of species analysed. **Table S6**. Statistics for dependent (D) and independent (I) evolution between pairs of 11 characters (Ch). Statistical (in)dependence tests by using Bayes Factors. **Table S7**. Z-score and transition rates between pairs of 11 characters (Ch) in dependent evolution. **Table S8**. Contingent evolutioin (C) detected within dependent evolution (D). Correspondence between dependent and contingent evolution are shown. Additional file 2: Table S2. Check list of all of species belonging to genera used in this study. Additional file 3:
**Supplementary Data S1.** The alignment, partitioning and substitution models for construction of phylogenetic tree.

## Abbreviations

NGP: Nymphalid ground plan
R: Root
B: Basal
Cp: Central proximal
Cd: Central distal
BOp: Border proximal
BOd: Border distal
DS: Discal spots
ESs: Eyespots
Ch: Character
MCMC: Markov chain Monte Carlo
