# Supplementary material for: Gradual and contingent evolutionary emergence of leaf mimicry in butterfly wing patterns
Source: BMC Evol Biol. 2014 Nov 25;14:229. doi: 10.1186/s12862-014-0229-5 (PMC4261531; doi:10.1186/s12862-014-0229-5)
Supplement: Additional file 1: Figure S1. — Phylogenetic relationship among genera of Kallima, Junoniini, and Nymphalini. Phylogeny of 22 genera of Nymphalinae is shown. Figure S2. Comparison of leaf wing patterns among three species of the genus Kallima. The ventral fore and hind wings of three species of Kallima are shown. Figure S3. Phylogenetic tree for Nymphalinae based on nine protein-coding gene sequences. The phylogenetic tree inferred by Bayesian analysis is depicted with branch lengths proportional to time. Figure S4. Contingent order detected as a bias of the transition rate using the Z-score. Contingency was analysed by comparing specific sets of transition parameters and the corresponding Z-scores. Table S1. Sampling density of species in this study. The genera of Nymphalinae and the number of species within the genera are described. Table S3. Species and Genbank accession numbers of the nine genes used. Eight nuclear (wingless, ef-1α, RpS5, GADPH, ArgKin, CAD, IDH and MDH) and one mitochondrial (cox1) gene accession IDs were listed. Table S4. The character states of Nymphalinae ventral-sided forewing patterns based on the Nymphalid ground plan used in this study. Character coding of forewing of species analysed. Table S5. The character states of Nymphalinae ventral-sided hindwing patterns based on the Nymphalid ground plan used in this study. Character coding of hindwing of species analysed. Table S6. Statistics for dependent (D) and independent (I) evolution between pairs of 11 characters (Ch). Statistical (in)dependence tests by using Bayes Factors. Table S7. Z-score and transition rates between pairs of 11 characters (Ch) in dependent evolution. Table S8. Contingent evolutioin (C) detected within dependent evolution (D). Correspondence between dependent and contingent evolution are shown. [file 12862_2014_229_MOESM1_ESM.pdf]

Gradual and contingent evolutionary emergence of leaf mimicry in butterfly wing patterns

Takao K Suzuki[1*], Shuichiro Tomita[1], Hideki Sezutsu[1]

[1] Transgenic Silkworm Research Unit, Genetically Modified Organism Research Center, National Institute of Agrobiological Sciences
1-2 Oowashi, Tsukuba, Ibaraki 305-8634 JAPAN

[*]Corresponding author
TKS: homaresuzuki@gmail.com

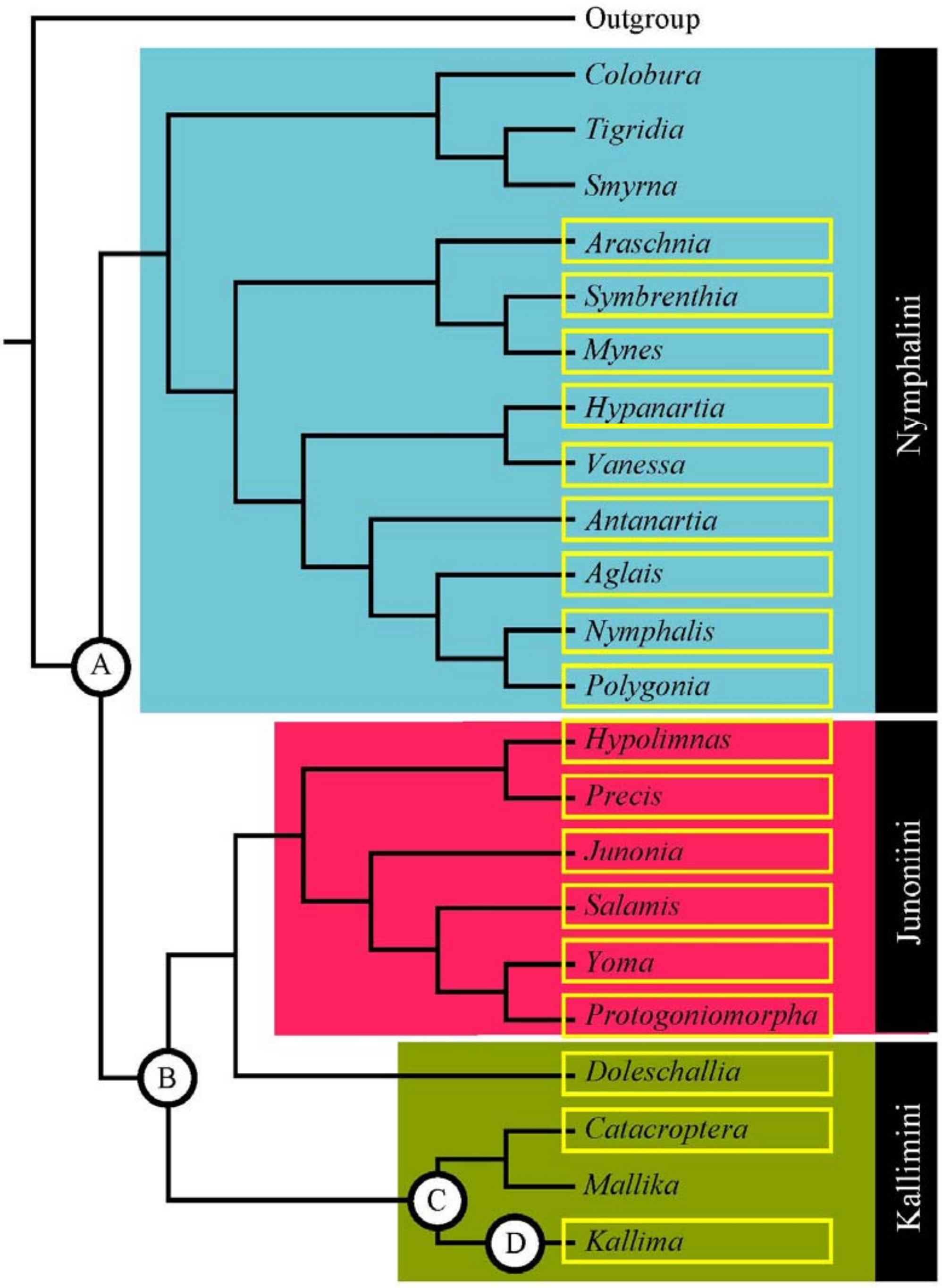

Supplementary Figure S1. Phylogenetic relationship among genera of *Kallima*, *Junoniini*, and *Nymphalini*. The phylogenetic relationship among all the genera included in the higher taxon is shown by Wahlberg [34–36]. The genera used in this study are indicated by yellow-colored squares. Ancestral character states were reconstructed on the nodes of the phylogeny (A–D).

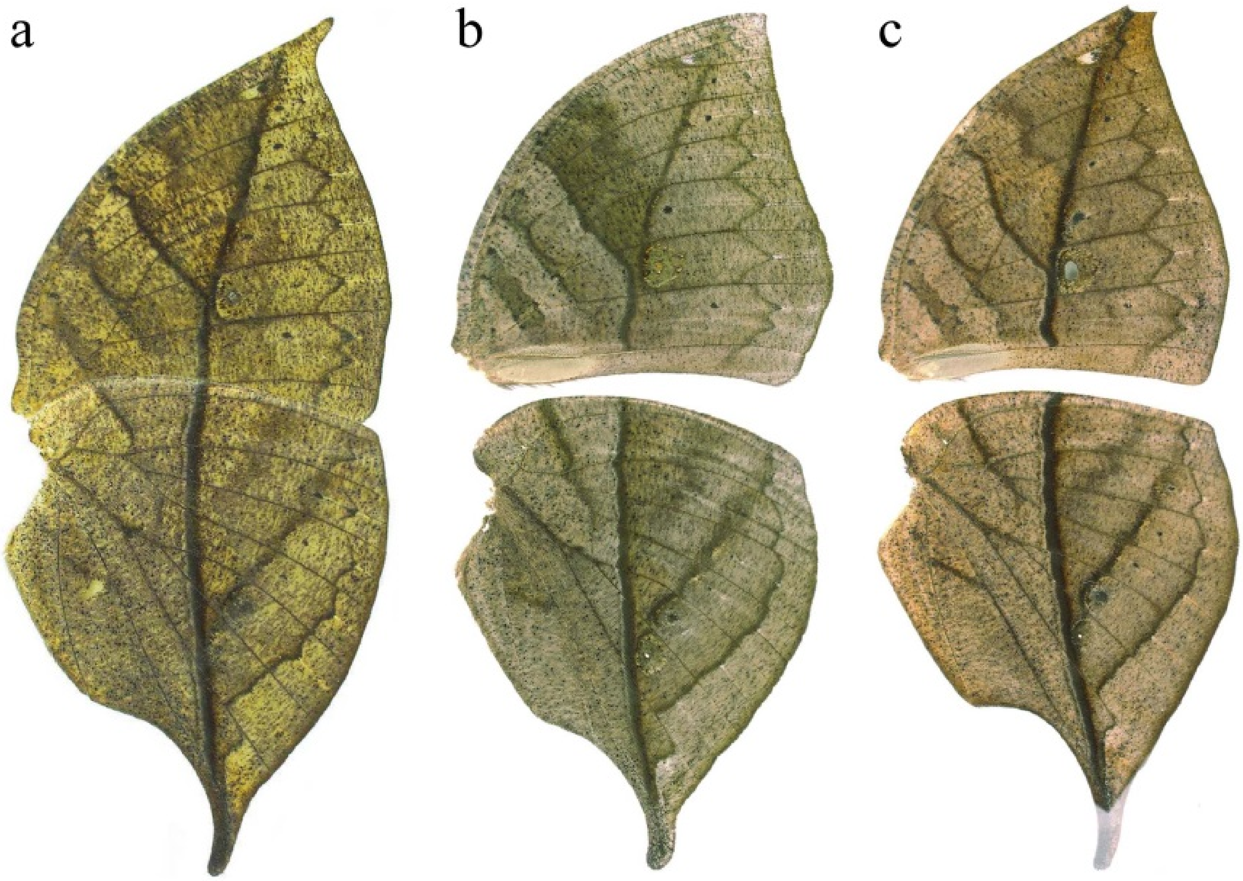

Supplementary Figure S2. Comparison of leaf wing patterns among three species of the genus Kallima. The ventral fore and hind wings of three species of *Kallima* are shown. These wing patterns, but not the pigmentation, appear to be almost identical. (a) *Kallima inachus*, (b) *Kallima paralekta*, (c) and *Kallima alompra*.

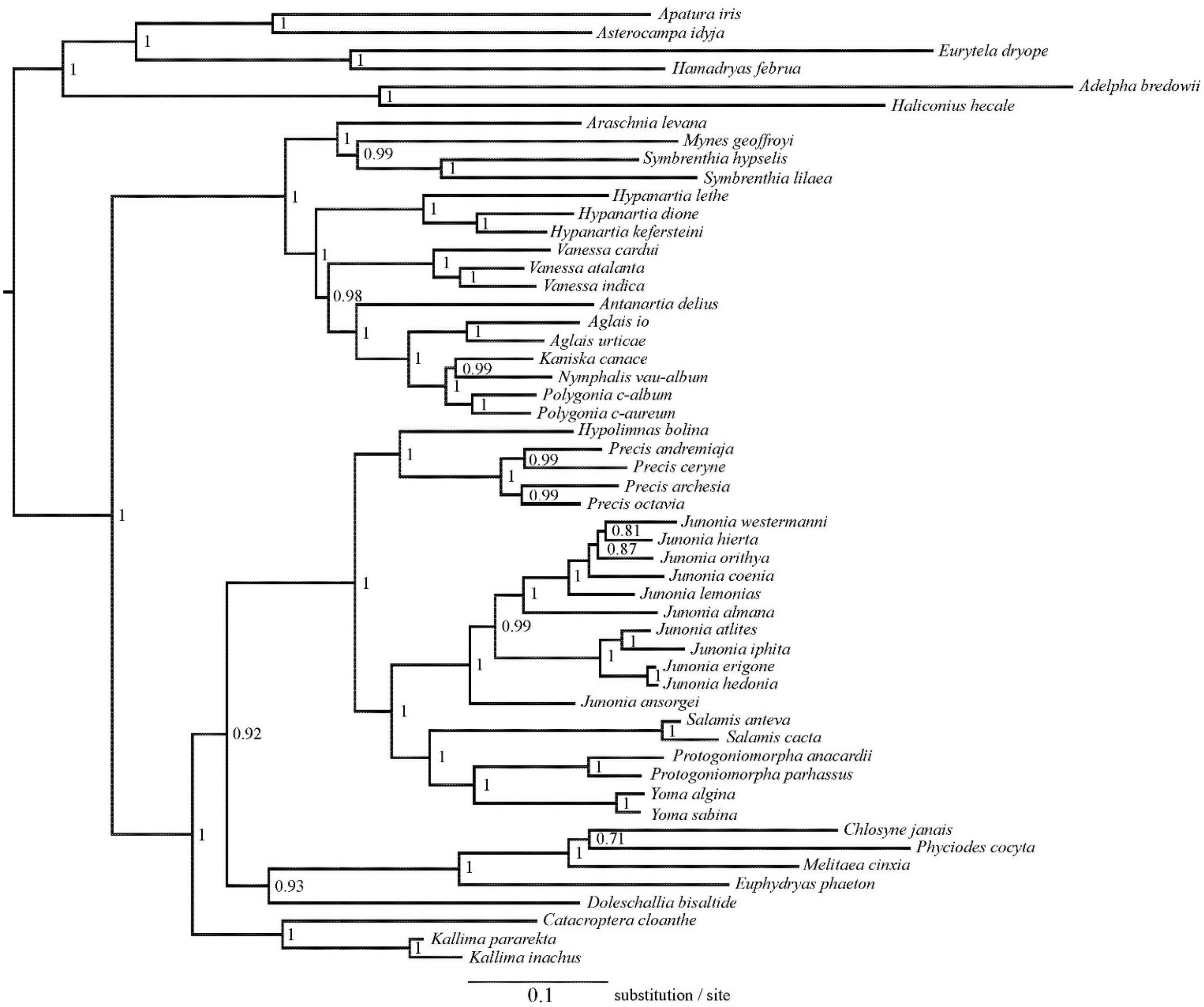

Supplementary Figure S3. Phylogenetic tree for Nymphalinae based on nine protein-coding gene sequences. The phylogenetic tree was inferred by Bayesian analysis. Posterior probability values are shown at each node. Branch lengths are proportional to time.

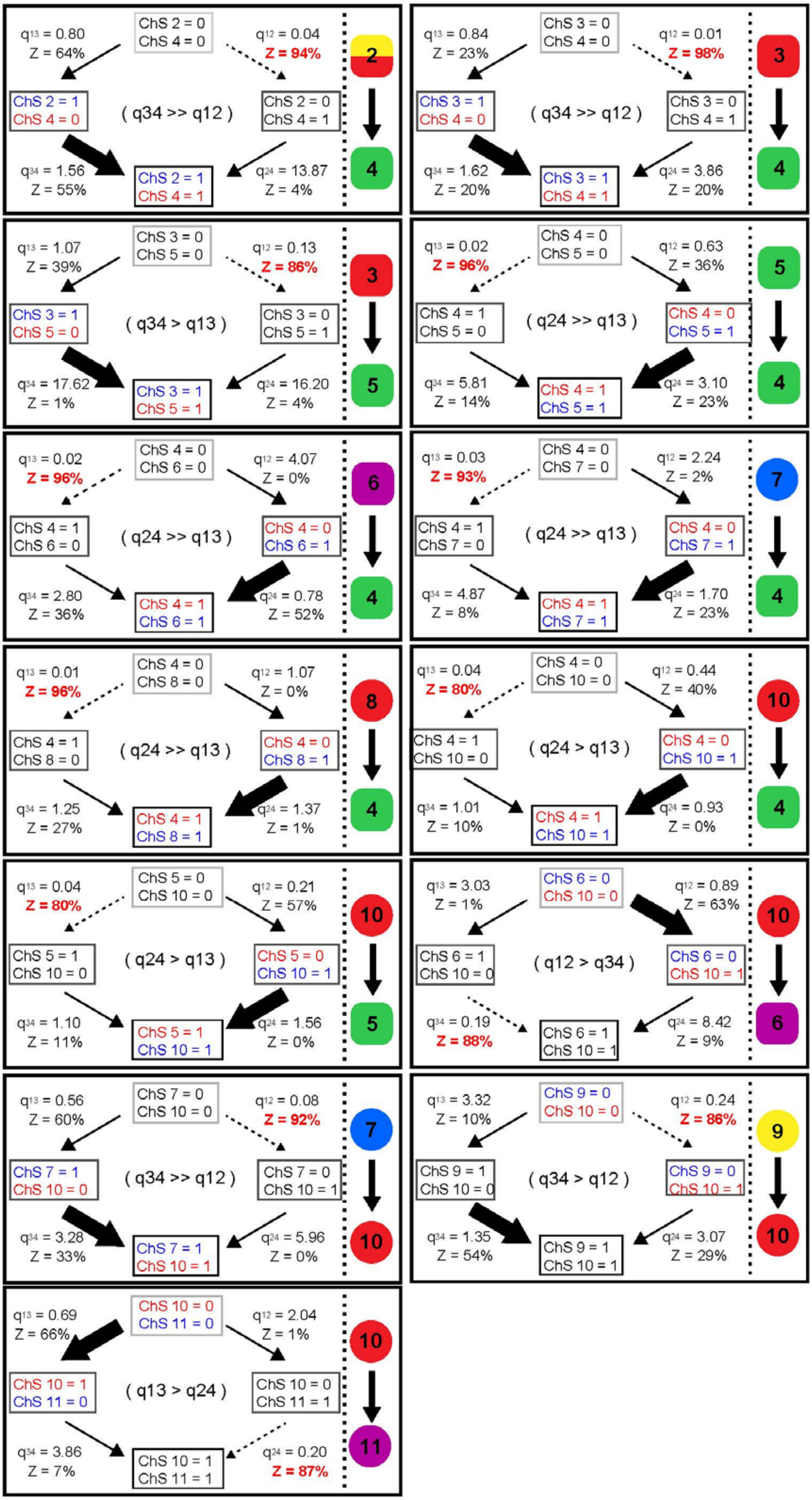
q13 = 0.80
Z = 64%
ChS 2 = 0
ChS 4 = 0
q12 = 0.04
Z = 94%
2
ChS 2 = 1
ChS 4 = 0
(q34 >> q12)
ChS 2 = 0
ChS 4 = 1
q34 = 1.56
Z = 55%
ChS 2 = 1
ChS 4 = 1
q24 = 13.87
Z = 4%
4
q13 = 0.84
Z = 23%
ChS 3 = 0
ChS 4 = 0
q12 = 0.01
Z = 98%
3
ChS 3 = 1
ChS 4 = 0
(q34 >> q12)
ChS 3 = 0
ChS 4 = 1
q34 = 1.62
Z = 20%
ChS 3 = 1
ChS 4 = 1
q24 = 3.86
Z = 20%
4
q13 = 1.07
Z = 39%
ChS 3 = 0
ChS 5 = 0
q12 = 0.13
Z = 86%
3
ChS 3 = 1
ChS 5 = 0
(q34 > q13)
ChS 3 = 0
ChS 5 = 1
q34 = 17.62
Z = 1%
ChS 3 = 1
ChS 5 = 1
q24 = 16.20
Z = 4%
5
q13 = 0.02
Z = 96%
ChS 4 = 0
ChS 5 = 0
q12 = 0.63
Z = 36%
5
ChS 4 = 1
ChS 5 = 0
(q24 >> q13)
ChS 4 = 0
ChS 5 = 1
q34 = 5.81
Z = 14%
ChS 4 = 1
ChS 5 = 1
q24 = 3.10
Z = 23%
4
q13 = 0.02
Z = 96%
ChS 4 = 0
ChS 6 = 0
q12 = 4.07
Z = 0%
6
ChS 4 = 1
ChS 6 = 0
(q24 >> q13)
ChS 4 = 0
ChS 6 = 1
q34 = 2.80
Z = 36%
ChS 4 = 1
ChS 6 = 1
q24 = 0.78
Z = 52%
4
q13 = 0.03
Z = 93%
ChS 4 = 0
ChS 7 = 0
q12 = 2.24
Z = 2%
7
ChS 4 = 1
ChS 7 = 0
(q24 >> q13)
ChS 4 = 0
ChS 7 = 1
q34 = 4.87
Z = 8%
ChS 4 = 1
ChS 7 = 1
q24 = 1.70
Z = 23%
4
q13 = 0.01
Z = 96%
ChS 4 = 0
ChS 8 = 0
q12 = 1.07
Z = 0%
8
ChS 4 = 1
ChS 8 = 0
(q24 >> q13)
ChS 4 = 0
ChS 8 = 1
q34 = 1.25
Z = 27%
ChS 4 = 1
ChS 8 = 1
q24 = 1.37
Z = 1%
4
q13 = 0.04
Z = 80%
ChS 4 = 0
ChS 10 = 0
q12 = 0.44
Z = 40%
10
ChS 4 = 1
ChS 10 = 0
(q24 > q13)
ChS 4 = 0
ChS 10 = 1
q34 = 1.01
Z = 10%
ChS 4 = 1
ChS 10 = 1
q24 = 0.93
Z = 0%
4
q13 = 0.04
Z = 80%
ChS 5 = 0
ChS 10 = 0
q12 = 0.21
Z = 57%
10
ChS 5 = 1
ChS 10 = 0
(q24 > q13)
ChS 5 = 0
ChS 10 = 1
q34 = 1.10
Z = 11%
ChS 5 = 1
ChS 10 = 1
q24 = 1.56
Z = 0%
5
q13 = 3.03
Z = 1%
ChS 6 = 0
ChS 10 = 0
q12 = 0.89
Z = 63%
10
ChS 6 = 1
ChS 10 = 0
(q12 > q34)
ChS 6 = 0
ChS 10 = 1
q34 = 0.19
Z = 88%
ChS 6 = 1
ChS 10 = 1
q24 = 8.42
Z = 9%
6
q13 = 0.56
Z = 60%
ChS 7 = 0
ChS 10 = 0
q12 = 0.08
Z = 92%
7
ChS 7 = 1
ChS 10 = 0
(q34 >> q12)
ChS 7 = 0
ChS 10 = 1
q34 = 3.28
Z = 33%
ChS 7 = 1
ChS 10 = 1
q24 = 5.96
Z = 0%
10
q13 = 3.32
Z = 10%
ChS 9 = 0
ChS 10 = 0
q12 = 0.24
Z = 86%
9
ChS 9 = 1
ChS 10 = 0
(q34 > q12)
ChS 9 = 0
ChS 10 = 1
q34 = 1.35
Z = 54%
ChS 9 = 1
ChS 10 = 1
q24 = 3.07
Z = 29%
10
q13 = 0.69
Z = 66%
ChS 10 = 0
ChS 11 = 0
q12 = 2.04
Z = 1%
10
ChS 10 = 1
ChS 11 = 0
(q13 > q24)
ChS 10 = 0
ChS 11 = 1
q34 = 3.86
Z = 7%
ChS 10 = 1
ChS 11 = 1
q24 = 0.20
Z = 87%
11

Supplementary Figure S4. Contingent order detected as a bias of the transition rate using the *Z*-score. Contingency was analysed by comparing specific sets of transition parameters and the corresponding *Z*-scores (i.e. q12 vs. q34; q13 vs. q24). Transitions with a lower probability of occurrence are shown by dashed arrows (*Z*-score >70%); the corresponding transitions with a higher probability of evolving are indicated by thick arrows. Thus, thick arrows highlight the contingent dependency of changes between some pairs of character states (ChS). In each panel, one character state change (red) is contingent upon the other character state change (blue). If a character state indicated in blue has a value of ‘1’, that state change precedes the corresponding character state change depicted in red. On the other hand, if a character state indicated in blue has a value of ‘0’, that state change follows the corresponding character state change depicted in red. Contingencies between pairs of character state changes are shown in the right panel of each box and are summarized as a network in Figure 4c.

Supplementary Table S1. Sampling density of species in this study. The description of genera and the number of species was obtained from the database of the Nymphalid systematics group (http://nymphalidae.utu.fi/index.htm).

Higher taxon	Genus	Number of species described	Number of species used in this study
Kallimini	*Kallima*	7	2
Kallimini	*Mallika*	1	X
Kallimini	*Catacroptera*	1	1
Kallimini	*Doleschallia*	9	1
Junoniini	*Protogoniomorpha*	2	2
Junoniini	*Yoma*	2	2
Junoniini	*Salamis*	3	2
Junoniini	*Junonia*	35	11
Junoniini	*Precis*	15	4
Junoniini	*Hypolimnas*	26	1
Nymphalini	*Polygonia*	16	2
Nymphalini	*Nymphalis*	5	2
Nymphalini	*Aglais*	5	2
Nymphalini	*Vanessa*	18	3
Nymphalini	*Antanartia*	5	1
Nymphalini	*Hypanartia*	14	3
Nymphalini	*Mynes*	10	1
Nymphalini	*Araschnia*	8	1
Nymphalini	*Symbrenthia*	15	2
Nymphalini	*Smyrna*	2	X
Nymphalini	*Tigridia*	1	X
Nymphalini	*Colobura*	2	X

Supplementary Table S3. Species and Genbank accession numbers of the nine genes used. For images of voucher specimens, see the NSG's DNA sequences database http://nymphalidae.utu.fi/db.php

Higher taxon	Species	*cox-I*	*ef-1α*	*wingless*	*RpS5*	*GAPDH*	*ArgKin*	*CAD*	*IDH*	*MDH*
Limenitidinae	*Adelpha bredowii*	AY788591	AY788693	AY788457	-	-	-	-	-	-
Heliconinae	*Heliconius hecale*	AY090202	AY090168	AY090135	EU141415	EU141514	EU141281	EU141337	EU141574	EU141638
Apaturinae	*Apatura iris*	AY090199	AY090165	AY090132	EU141413	EU141513	EU141280	EU141335	EU141572	EU141636
Apaturinae	*Asterocampa idyja*	GQ864738	GQ864426	GQ864426	GQ865397	GQ864930	GQ864518	GQ864613	GQ865067	GQ865175
Biblidinae	*Eurytela dryope*	AY218242	AY218262	AY218280	GQ865441	GQ864971	-	GQ864654	GQ865099	GQ865215
Biblidinae	*Hamadryas februa*	AY090216	AY090182	AY090149	EU141402	-	EU141271	EU141324	EU141561	EU141625
Nymphalini	*Araschnia levana*	AY248780	GQ864829	GQ864423	GQ865391	GQ864925	GQ864516	GQ864610	GQ865064	GQ865171
Nymphalini	*Mynes geoffroyi*	AY248778	AY248803	AF412760	EU141405	EU141505	EU141273	EU141327	EU141564	EU141628
Nymphalini	*Symbrenthia hypselis*	AY788680	AY788818	AY788578	-	-	-	-	-	-
Nymphalini	*Symbrenthia lilaea*	AY788679	AY788817	AY788577	GQ865497	GQ865034	GQ864582	GQ864713	GQ865150	GQ865266
Nymphalini	*Hypanartia lethe*	AF187774	AY788760	AY788520	HQ735056	HQ734980	HQ734859	HQ734880	HQ735001	HQ735022
Nymphalini	*Hypanartia dione*	HQ734933	HQ734953	HQ734843	HQ735060	HQ734984	HQ734862	HQ734884	HQ735004	HQ735026
Nymphalini	*Hypanartia kefersteini*	AY788640	AY788759	AY788519	HQ735061	HQ734985	HQ734863	HQ734885	HQ735005	HQ735027
Nymphalini	*Vanessa cardui*	HQ734922	HQ734936	HQ734838	HQ735047	HQ734971	HQ734851	HQ734873	HQ734993	HQ735015
Nymphalini	*Vanessa atalanta*	HQ734919	AY090187	AF412772	GQ865508	GQ865045	GQ864589	GQ864722	GQ865155	GQ865275
Nymphalini	*Vanessa indica*	HQ734916	AY788825	AY788585	HQ735045	HQ734969	HQ734849	HQ734871	HQ734991	HQ735013
Nymphalini	*Antanartia delius*	AY788610	AY788712	AY788473	GQ865387	GQ864922	GQ864513	GQ864606	GQ865062	GQ865167
Nymphalini	*Aglais io*	AY248785	AY248810	AF412766	FJ639576	FJ639521	-	-	-	-
Nymphalini	*Aglais urticae*	AY248786	AY248811	AF412777	FJ639577	FJ639522	GQ864509	GQ864599	GQ865055	GQ865161
Nymphalini	*Kanisca canace*	FJ639397	FJ639493	AY248833	FJ639584	FJ639529	-	-	-	-
Nymphalini	*Nymphalis vau-album*	AY248791	AY248816	AY248832	FJ639581	FJ639526	-	-	-	-
Nymphalini	*Polygonia c-album*	JN093202	AY090188	AY090154	FJ639569	FJ639514	-	HQ734879	HQ735006	HQ735028
Nymphalini	*Polygonia c-aureum*	FJ639403	AY248824	FJ639347	FJ639568	FJ639513	GQ864575	GQ864701	GQ865140	GQ865257
Junoniini	*Hypolimnas bolina*	AY090224	AY090190	AY090156	-	-	-	-	-	-
Junoniini	*Precis andremiaja*	AY788664	AY788802	AY788562	-	-	-	-	-	-
Junoniini	*Precis ceryne*	AY788667	AY788805	AY788565	-	-	-	-	-	-
Junoniini	*Precis archesia*	AY788666	AY788804	AY788564	-	-	-	-	-	-
Junoniini	*Precis octavia*	AY788669	AY788807	AY788567	-	-	-	-	-	-
Junoniini	*Junonia westermanni*	EU053319	EU053356	EU053393	-	-	-	-	-	-
Junoniini	*Junonia hierta*	EU053305	EU053344	EU053381	-	-	-	-	-	-
Junoniini	*Junonia orithya*	EU053315	EU053351	EU053389	-	-	-	-	-	-
Junoniini	*Junonia coenia*	AY788643	AY248801	AY248826	-	-	-	-	-	-
Junoniini	*Junonia lemonias*	EU053309	EU053346	EU053383	-	-	-	-	-	-
Junoniini	*Junonia alamana*	EU053289	EU053323	EU053360	-	-	-	-	-	-
Junoniini	*Junonia atlites*	EU053294	EU053330	EU053366	-	-	-	-	-	-
Junoniini	*Junonia iphita*	AY090225	AY090191	AY090157	-	-	-	-	-	-
Junoniini	*Junonia erigone*	AY788644	AY788763	AY788523	-	-	-	-	-	-
Junoniini	*Junonia hedonia*	EU053303	EU053340	EU053377	-	-	-	-	-	-
Junoniini	*Junonia ansorgei*	EU053290	EU053326	EU053363	-	-	-	-	-	-
Junoniini	*Salamis anteva*	AY788675	AY788813	AY788573	-	-	-	-	-	-
Junoniini	*Salamis cacta*	AY788676	AY788814	AY788574	-	-	-	-	-	-
Junoniini	*Protogoniomorpha anacardii*	AY090223	AY090189	AY090155	-	-	-	-	-	-
Junoniini	*Protogoniomorpha parhassus*	AY788673	AY788811	AY788571	-	-	-	-	-	-
Junoniini	*Yoma algina*	AY788692	AY788830	AY788590	-	-	-	-	-	-
Junoniini	*Yoma sabina*	EU053399	EU053397	EU053398	-	-	-	-	-	-
Melitaeini	*Chlosyne janais*	AY788620	AY788730	AY788491	GQ865414	GQ864943	GQ864533	GQ864629	-	GQ865190
Melitaeini	*Phyciodes cocyta*	AY156607	AY090192	AY090158	GQ865486	-	GQ864571	GQ864697	-	GQ865253
Melitaeini	*Melitaea cinxia*	AY788656	AY788776	AY788536	EU141420	EU141518	EU141284	EU141342	EU141579	EU141643
Melitaeini	*Euphydryas phaeton*	AF187797	AY788747	AY788508	GQ865434	GQ864965	GQ864546	GQ864647	-	GQ865208
Kallimini	*Doleschallia bisaltide*	AY788621	AY788735	AY788496	GQ865423	GQ864955	GQ864539	-	GQ865086	GQ865199
Kallimini	*Catacroptera cloanthe*	AY788619	AY788724	AY788485	GQ865409	GQ864939	GQ864528	GQ864625	-	GQ865186
Kallimini	*Kallima paralekta*	AY090229	AY090197	AY090163	EU141404	EU141504	EU141272	EU141326	EU141563	EU141627
Kallimini	*Kallima inachus*	AY788650	AY788769	AY788529	-	-	-	-	-	-

Suppelementary Table S4. The character states of Nymphalinae ventral-sided forewing patterns based on the Nymphalid ground plan used in this study

	character 1	character 2	character 3	character 4	character 5	character 6
	Parallelism of DS & B	Attachment of DS & Cp	A single broken straight line of Cd	Bending direction of BOp	Straightness of upper side of BOp	Vestigiality of ESs
Adelpha bredowii	parallel	not attached	not a single broken line	bending proximally	not straight	vestigial
Heliconius hecale	parallel	not attached	not a single broken line	bending proximally	not straight	vestigial
Apatura iris	parallel	not attached	not a single broken line	bending proximally	not straight	not vestigial
Asterocampa idyja	parallel	not attached	not a single broken line	bending proximally	not straight	not vestigial
Eurytela dryope	parallel	not attached	not a single broken line	bending proximally	not straight	vestigial
Hamadryas februa	parallel	not attached	not a single broken line	bending proximally	not straight	not vestigial
Araschnia levana	parallel	not attached	not a single broken line	bending proximally	not straight	not vestigial
Mynes geoffroyi	not parallel / parallel	not attached	not a single broken line	bending proximally	not straight	vestigial
Symbrenthia hypselis	parallel	not attached	not a single broken line	bending proximally	not straight	not vestigial
Symbrenthia lilaea	parallel	not attached	not a single broken line	bending proximally	not straight	not vestigial
Hypanartia lethe	parallel	not attached	not a single broken line	bending proximally	not straight	vestigial
Hypanartia dione	parallel	not attached	not a single broken line	bending proximally	not straight	not vestigial
Hypanartia kefersteini	parallel	not attached	not a single broken line	bending proximally	not straight	not vestigial
Vanessa cardui	parallel	not attached	not a single broken line	bending proximally	not straight	not vestigial
Vanessa atalanta	parallel	not attached	not a single broken line	bending proximally	not straight	not vestigial
Vanessa indica	parallel	not attached	not a single broken line	bending proximally	not straight	not vestigial
Antanartia delius	parallel	attached	not a single broken line	bending proximally	not straight	not vestigial
Aglais io	parallel	not attached	not a single broken line	bending proximally	not straight	vestigial
Aglais urticae	parallel	attached	not a single broken line	bending proximally	not straight	vestigial
Kanisca canace	not parallel	not attached	not a single broken line	bending proximally	not straight	vestigial
Nymphalis vau-album	not parallel	not attached	not a single broken line	bending proximally	not straight	vestigial
Polygonia c-album	not parallel	attached	not a single broken line / a single broken line	bending proximally	not straight	vestigial
Polygonia c-aureum	not parallel	attached	not a single broken line	bending proximally	not straight	vestigial
Hypolimnas bolina	parallel	not attached	not a single broken line	bending proximally	not straight	not vestigial
Precis andremiaja	parallel	not attached	not a single broken line	bending proximally	not straight	not vestigial
Precis ceryne	not parallel / parallel	not attached	not a single broken line	bending proximally	not straight / straight	not vestigial
Precis archesia	parallel	not attached / attached	a single broken line	bending proximally	straight	not vestigial
Precis octavia	parallel	not attached / attached	not a single broken line	bending proximally	not straight / straight	not vestigial
Junonia westermanni	parallel	not attached	not a single broken line	bending proximally	not straight	vestigial
Junonia hierta	parallel	not attached	not a single broken line	bending proximally	not straight	not vestigial
Junonia orithya	parallel	not attached	not a single broken line	bending proximally	not straight	not vestigial
Junonia coenia	parallel	not attached	a single broken line	bending proximally	not straight	not vestigial
Junonia lemonias	parallel	attached	not a single broken line	bending proximally	not straight	not vestigial
Junonia alamana	parallel	not attached	not a single broken line / a single broken line	bending proximally	not straight / straight	not vestigial / vestigial
Junonia atlites	parallel	attached	not a single broken line	bending proximally	not straight	not vestigial
Junonia iphita	parallel	attached	not a single broken line	bending proximally	not straight	not vestigial
Junonia erigone	parallel	attached	not a single broken line	bending proximally	not straight	not vestigial
Junonia hedonia	parallel	attached	not a single broken line	bending proximally	not straight	not vestigial
Junonia ansorgei	parallel	not attached	not a single broken line	bending proximally	not straight	not vestigial
Salamis anteva	parallel	not attached	not a single broken line	bending proximally	not straight	vestigial
Salamis cacta	parallel	not attached	not a single broken line	bending proximally	not straight	vestigial
Protogoniomorpha anacardii	parallel	not attached	not a single broken line	bending proximally	not straight	vestigial
Protogoniomorpha parhassus	parallel	not attached	not a single broken line	bending proximally	not straight	not vestigial
Yoma algina	parallel	not attached	not a single broken line	bending proximally	not straight / straight	not vestigial
Yoma sabina	parallel	not attached	not a single broken line / a single broken line	bending proximally	not straight / straight	vestigial
Chlosyne janais	parallel	not attached	not a single broken line	bending proximally	not straight	not vestigial
Phyciodes cocyta	parallel	not attached	not a single broken line	bending proximally	not straight	not vestigial
Melitaea cinxia	parallel	not attached	not a single broken line	bending proximally	not straight	vestigial
Euphydryas phaeton	parallel	not attached	not a single broken line	bending proximally	not straight	vestigial
Doleschallia bisaltide	parallel	not attached	a single broken line	bending proximally	straight	vestigial
Catacroptera cloanthe	parallel	not attached	a single broken line	bending proximally	not straight	not vestigial
Kallima paralekta	parallel	attached	a single broken line	bending distally	straight	vestigial
Kallima inachus	parallel	attached	a single broken line	bending distally	straight	vestigial

Supplementary Table S5. The character states of Nymphalinae ventral-sided hindwing patterns based on the Nymphalid ground plan used in this study

	character 7	character 8	character 9	character 10	character 11
	**Vestigiality of B**	**Fragmentation of Cp**	**Vestigiality of DS**	**Straightness of Cd**	**Vestigiality of ESs**
Adelpha bredowii	not vestigial	not fragmentation	not vestigial	not straight	vestigial
Heliconius hecale	vestigial	not fragmentation	vestigial	not straight	vestigial
Apatura iris	vestigial	not fragmentation	vestigial	not straight	not vestigial
Asterocampa idyja	not vestigial	not fragmentation	not vestigial	not straight	not vestigial
Eurytela dryope	not vestigial / vestigial	not fragmentation	not vestigial / vestigial	not straight	not vestigial / vestigial
Hamadryas februa	vestigial	not fragmentation	not vestigial	not straight	not vestigial
Araschnia levana	not vestigial	not fragmentation	vestigial	not straight	not vestigial
Mynes geoffroyi	vestigial	not fragmentation	vestigial	not straight	vestigial
Symbrenthia hypselis	not vestigial	fragmentation	vestigial	not straight	not vestigial
Symbrenthia lilaea	not vestigial	fragmentation	vestigial	not straight	not vestigial
Hypanartia lethe	not vestigial	not fragmentation	not vestigial	not straight	not vestigial
Hypanartia dione	not vestigial	fragmentation	not vestigial	not straight	not vestigial
Hypanartia kefersteini	not vestigial	not fragmentation	not vestigial	not straight	not vestigial
Vanessa cardui	not vestigial	not fragmentation	not vestigial	not straight	not vestigial
Vanessa atalanta	not vestigial	not fragmentation	not vestigial	not straight	not vestigial
Vanessa indica	not vestigial	not fragmentation	not vestigial	not straight	not vestigial
Antanartia delius	not vestigial	not fragmentation	not vestigial	not straight	not vestigial
Aglais io	not vestigial	not fragmentation	not vestigial	not straight	vestigial
Aglais urticae	not vestigial	not fragmentation	not vestigial	not straight	vestigial
Kanisca canace	not vestigial	not fragmentation	not vestigial	not straight	vestigial
Nymphalis vau-album	not vestigial	not fragmentation	not vestigial	not straight	vestigial
Polygonia c-album	not vestigial	not fragmentation	not vestigial	not straight	vestigial
Polygonia c-aureum	not vestigial	not fragmentation	not vestigial	not straight	vestigial
Hypolimnas bolina	vestigial	not fragmentation	vestigial	not straight / straight	not vestigial / vestigial
Precis andremiaja	not vestigial	not fragmentation	not vestigial	not straight	not vestigial
Precis ceryne	not vestigial / vestigial	not fragmentation	not vestigial	straight	not vestigial
Precis archesia	not vestigial / vestigial	not fragmentation	not vestigial / vestigial	straight	not vestigial / vestigial
Precis octavia	not vestigial	not fragmentation	not vestigial / vestigial	not straight	not vestigial / vestigial
Junonia westermanni	not vestigial	not fragmentation	not vestigial / vestigial	not straight	not vestigial
Junonia hierta	not vestigial	not fragmentation	vestigial	not straight	vestigial
Junonia orithya	not vestigial	not fragmentation	not vestigial	not straight	not vestigial / vestigial
Junonia coenia	not vestigial / vestigial	fragmentation	not vestigial / vestigial	not straight	not vestigial / vestigial
Junonia lemonias	not vestigial	not fragmentation	not vestigial	not straight	not vestigial / vestigial
Junonia alamana	not vestigial / vestigial	not fragmentation	not vestigial / vestigial	straight	not vestigial / vestigial
Junonia atlites	not vestigial	fragmentation	not vestigial	not straight	not vestigial
Junonia iphita	not vestigial	not fragmentation	not vestigial	not straight	not vestigial
Junonia erigone	not vestigial	not fragmentation	not vestigial	not straight	not vestigial
Junonia hedonia	not vestigial	not fragmentation	not vestigial / vestigial	not straight	not vestigial
Junonia ansorgei	vestigial	not fragmentation	vestigial	straight	not vestigial
Salamis anteva	not vestigial	not fragmentation	vestigial	not straight	vestigial
Salamis cacta	not vestigial	not fragmentation	vestigial	not straight	vestigial
Protogoniomorpha anacardii	not vestigial	not fragmentation	vestigial	straight	not vestigial
Protogoniomorpha parhassus	not vestigial	not fragmentation	vestigial	straight	not vestigial
Yoma algina	vestigial	not fragmentation	vestigial	straight	not vestigial / vestigial
Yoma sabina	not vestigial	not fragmentation	vestigial	straight	vestigial
Chlosyne janais	not vestigial	fragmentation	not vestigial	not straight	not vestigial
Phyciodes cocyta	not vestigial	not fragmentation	vestigial	not straight	not vestigial
Melitaea cinxia	not vestigial	not fragmentation	not vestigial	not straight	not vestigial
Euphydryas phaeton	not vestigial	not fragmentation	not vestigial	not straight	vestigial
Doleschallia bisaltide	not vestigial	not fragmentation	vestigial	straight	not vestigial
Catacroptera cloanthe	vestigial	not fragmentation / fragmentation	vestigial	straight	not vestigial
Kallima paralekta	vestigial	fragmentation	vestigial	straight	vestigial
Kallima inachus	vestigial	fragmentation	vestigial	straight	vestigial

Supplementary Table S6. Statistics for dependent (D) and independent (I) evolution between pairs of 11 characters (Ch). *1 The median value of at least three replicates is shown. *2 Logarithmic Bayes Factors (log-BF) >2 are considered as positive evidence that two models differ. * Values that indicate the presence of differences between models are followed by an asterisk.

		log harmonic mean (D) *1	log harmonic mean (I) *1	log-BF *2
Ch 1	Ch 2	-32.66	-34.99	4.67*
Ch 1	Ch 3	-30.20	-30.13	-0.15
Ch 1	Ch 4	-14.64	-14.55	-0.19
Ch 1	Ch 5	-25.49	-25.36	-0.27
Ch 1	Ch 6	-40.81	-44.07	6.52*
Ch 1	Ch 7	-38.77	-38.38	-0.78
Ch 1	Ch 8	-33.37	-33.19	-0.36
Ch 1	Ch 9	-41.01	-41.53	1.05
Ch 1	Ch 10	-30.21	-29.51	-1.40
Ch 1	Ch 11	-34.90	-36.83	3.86*
Ch 2	Ch 3	-44.71	-44.66	-0.11
Ch 2	Ch 4	-29.91	-32.15	4.48*
Ch 2	Ch 5	-41.27	-41.08	-0.37
Ch 2	Ch 6	-56.42	-55.93	-0.98
Ch 2	Ch 7	-50.98	-50.40	-1.15
Ch 2	Ch 8	-48.53	-48.58	0.10
Ch 2	Ch 9	-56.71	-56.13	-1.16
Ch 2	Ch 10	-50.40	-50.02	-0.76
Ch 2	Ch 11	-51.80	-51.72	-0.16
Ch 3	Ch 4	-26.14	-27.89	3.50*
Ch 3	Ch 5	-32.43	-36.64	8.43*
Ch 3	Ch 6	-52.52	-52.43	-0.18
Ch 3	Ch 7	-48.22	-48.47	0.50
Ch 3	Ch 8	-44.99	-44.11	-1.76
Ch 3	Ch 9	-52.40	-52.72	0.65
Ch 3	Ch 10	-39.97	-43.51	7.07*
Ch 3	Ch 11	-47.40	-47.71	0.60
Ch 4	Ch 5	-21.57	-23.89	4.62*
Ch 4	Ch 6	-38.66	-40.09	2.85*
Ch 4	Ch 7	-35.29	-36.87	3.15*
Ch 4	Ch 8	-30.16	-31.77	3.24*
Ch 4	Ch 9	-39.97	-40.61	1.28
Ch 4	Ch 10	-31.24	-32.53	2.58*
Ch 4	Ch 11	-34.27	-35.10	1.66
Ch 5	Ch 6	-49.42	-48.99	-0.86
Ch 5	Ch 7	-44.93	-44.91	-0.05
Ch 5	Ch 8	-40.81	-40.77	-0.08
Ch 5	Ch 9	-49.30	-48.88	-0.84
Ch 5	Ch 10	-33.09	-39.58	12.98*
Ch 5	Ch 11	-44.21	-44.63	0.84
Ch 6	Ch 7	-59.74	-59.60	-0.29
Ch 6	Ch 8	-55.59	-55.46	-0.27
Ch 6	Ch 9	-63.34	-63.45	0.23
Ch 6	Ch 10	-56.30	-57.86	3.12*
Ch 6	Ch 11	-51.91	-60.16	16.50*
Ch 7	Ch 8	-52.51	-52.10	-0.82
Ch 7	Ch 9	-59.15	-61.29	4.29*
Ch 7	Ch 10	-46.00	-49.97	7.93*
Ch 7	Ch 11	-56.52	-55.85	-1.33
Ch 8	Ch 9	-57.80	-57.53	-1.43
Ch 8	Ch 10	-46.42	-46.16	-0.51
Ch 8	Ch 11	-51.87	-51.85	-0.04
Ch 9	Ch 10	-54.26	-57.55	6.57*
Ch 9	Ch 11	-60.97	-61.45	0.96
Ch 10	Ch 11	-51.26	-52.41	2.31*

Supplementary Table S7. ***Z*** **-score and transition rates between pairs of 11 characters (Ch) in dependent evolution.**

			q12	q13	q21	q24	q31	q34	q42	q43
Ch 1	Ch 2	transition rate	1.61	1.26	1.82	1.37	0.12	0.88	1.46	1.93
		Z -score (%)	6	14	3	9	62	0	4	3
Ch 1	Ch 6	transition rate	4.31	4.49	1.27	4.13	0.07	4.03	1.40	5.11
		Z -score (%)	8	9	56	12	83	0	6	5
Ch 1	Ch 11	transition rate	3.98	4.03	1.06	3.68	0.04	2.19	1.98	3.48
		Z -score (%)	8	12	59	12	89	0	2	17
Ch 2	Ch 4	transition rate	0.04	0.80	12.02	13.87	12.34	1.56	5.58	12.45
		Z -score (%)	94	64	8	4	0	55	39	2
Ch 3	Ch 4	transition rate	0.01	0.84	3.11	3.86	4.97	1.62	1.48	4.17
		Z -score (%)	98	23	34	20	12	20	63	22
Ch 3	Ch 5	transition rate	0.13	1.07	15.21	16.20	19.43	17.62	3.38	15.06
		Z -score (%)	86	39	7	4	1	1	56	2
Ch 3	Ch 10	transition rate	0.26	0.38	1.73	1.58	1.11	1.07	0.71	0.77
		Z -score (%)	37	3	0	0	9	8	27	17
Ch 4	Ch 5	transition rate	0.63	0.02	7.50	3.10	4.89	5.81	6.02	2.37
		Z -score (%)	36	96	6	23	26	14	18	56
Ch 4	Ch 6	transition rate	4.07	0.02	3.93	0.78	4.36	2.80	4.35	1.16
		Z -score (%)	0	96	19	52	21	36	11	72
Ch 4	Ch 7	transition rate	2.24	0.03	6.76	1.70	4.99	4.87	6.19	1.72
		Z -score (%)	2	93	1	23	8	8	3	54
Ch 4	Ch 8	transition rate	1.07	0.01	1.72	1.37	1.16	1.25	1.18	0.55
		Z -score (%)	0	96	16	1	40	27	37	66
Ch 4	Ch 10	transition rate	0.44	0.04	1.60	0.93	1.01	1.01	1.00	0.78
		Z -score (%)	40	80	2	0	10	10	13	27
Ch 5	Ch 10	transition rate	0.21	0.04	1.74	1.56	1.14	1.10	1.05	0.82
		Z -score (%)	57	80	0	0	8	11	14	26
Ch 6	Ch 10	transition rate	0.89	3.03	10.10	8.42	1.19	0.19	12.77	2.15
		Z -score (%)	63	1	0	9	74	88	1	33
Ch 6	Ch 11	transition rate	4.70	5.24	34.51	18.15	33.80	6.82	0.94	0.83
		Z -score (%)	5	0	3	8	3	30	82	82
Ch 7	Ch 9	transition rate	3.52	1.68	7.24	8.28	10.41	9.39	9.52	3.45
		Z -score (%)	11	26	1	1	2	4	2	22
Ch 7	Ch 10	transition rate	0.08	0.56	5.66	5.96	3.77	3.28	5.55	4.48
		Z -score (%)	92	60	2	0	20	33	7	4
Ch 9	Ch 10	transition rate	0.24	3.32	5.73	3.07	5.31	1.35	2.04	3.82
		Z -score (%)	86	10	1	29	1	54	30	5
Ch 10	Ch 11	transition rate	2.04	0.69	0.57	0.20	7.28	3.86	3.63	12.84
		Z -score (%)	1	66	81	87	0	7	17	3

Supplementary Table S8. Contingent evolutioin (C) detected within dependent evolution (D)

Dependent evolution

	Ch 1	Ch 2	Ch 3	Ch 4	Ch 5	Ch 6	Ch 7	Ch 8	Ch 9	Ch 10	Ch 11
Ch 1											
Ch 2	D										
Ch 3	—	—									
Ch 4	—	D	D								
Ch 5	—	—	D	D							
Ch 6	D	—	—	D	—						
Ch 7	—	—	—	D	—	—					
Ch 8	—	—	—	D	—	—	—				
Ch 9	—	—	—	—	—	—	D	—			
Ch 10	—	—	D	D	D	D	D	—	D		
Ch 11	D	—	—	—	—	D	—	—	—	D	

Contingent evolution

	Ch 1	Ch 2	Ch 3	Ch 4	Ch 5	Ch 6	Ch 7	Ch 8	Ch 9	Ch 10	Ch 11
Ch 1											
Ch 2	—										
Ch 3	—	—									
Ch 4	—	C	C								
Ch 5	—	—	C	C							
Ch 6	—	—	—	C	—						
Ch 7	—	—	—	C	—	—					
Ch 8	—	—	—	C	—	—	—				
Ch 9	—	—	—	—	—	—	—	—			
Ch 10	—	—	C	C	C	C	C	—	C		
Ch 11	—	—	—	—	—	—	—	—	—	C	
